# Electrophysiological Response to the Informative Value of Feedback Revealed in a Segmented Wisconsin Card Sorting Test

**DOI:** 10.3389/fpsyg.2018.00057

**Published:** 2018-02-05

**Authors:** Fuhong Li, Jing Wang, Bin Du, Bihua Cao

**Affiliations:** ^1^Advanced Research Institute, Chengdu University, Chengdu, China; ^2^School of Psychology, Jiangxi Normal University, Nanchang, China; ^3^School of Psychology, Southwest University, Chongqing, China; ^4^School of Psychology, Liaoning Normal University, Dalian, China

**Keywords:** feedback, rule acquisition, informative value, valence, P300

## Abstract

Feedback has two main components. One is valence that indicates the wrong or correct behavior, and the other is the informative value that refers to what we can learn from feedback. Aimed to explore the neural distinction of these two components, we provided participants with a segmented Wisconsin Card Sorting Task, in which they received either positive or negative feedback at different steps. The informative value was manipulated in terms of the order of feedback presentation. The results of event-related potentials time-locked to the feedback presentation confirmed that valence of feedback was processed in a broad epoch, especially in the time window of feedback-related negativity (FRN), reflecting detection of correct or wrong card sorting behavior. In contrast, the informative value of positive and negative feedback was mainly processed in the P300, possibly reflecting information updating or hypothesis revision. These findings provide new evidence that informative values of feedback are processed by cognitive systems that differ from those of feedback valence.

## Introduction

Human use feedback information to make rapid adjustments and optimize behavior. Feedback has two main components, valence^1^ and informative value (Özyurt et al., 2012; Tricomi and Fiez, 2012). The former specifies whether the current behavior is right or wrong, and the latter refers to the information that we can extract from the feedback and use in adjusting behavior (Zanolie et al., 2008; Mies et al., 2011; Lange et al., 2015).

Imaging studies on feedback valence have confirmed that the medial prefrontal cortex, including the anterior cingulate cortex (ACC), was more sensitive to negative feedback as compared with positive feedback (Tricomi et al., 2006; Van Leijenhorst et al., 2006; Van Duijvenvoorde et al., 2008; Dobryakova and Tricomi, 2013; Li et al., 2013; Gu et al., 2017). Researchers using event-related potentials (ERPs) have identified a component that is sensitive to the valence of feedback (Yeung et al., 2004; Boksem et al., 2009; Wild-Wall et al., 2009; Ferdinand et al., 2016). This feedback-related negativity (FRN) shows a relatively negative deflection following losses or negative feedback compared with wins or positive feedback (Nieuwenhuis et al., 2005; Van Der Veen et al., 2008; Hämmerer et al., 2011; Opitz et al., 2011; Arbel et al., 2013). The FRN peaks at around 300 ms and is maximal at fronto-central scalp electrode sites (Holroyd et al., 2003; Hajcak et al., 2005; Sato et al., 2005). However, when valence was weakened and the other related components, such as expectancy, were emphasized, FRN was no longer sensitive to feedback valence but was sensitive to expectancy (Oliveira et al., 2007; Ferdinand et al., 2012; Ferdinand and Opitz, 2014; Pfabigan et al., 2015).

Some studies have shown that the feedback valence is also associated with P300 (Hajcak et al., 2005; Groen et al., 2007; Yeung et al., 2005), but other investigators indicated that P300 is more likely associated with higher order cognition (Yeung and Sanfey, 2004; Sato et al., 2005; Hajcak et al., 2007; Bellebaum and Daum, 2008; Ferdinand et al., 2012). It has been found that the P300 (but not the FRN) may be related to task relevance (Ferdinand et al., 2012; Ferdinand and Kray, 2013) or behavioral adjustment (Yeung and Sanfey, 2004). For example, in a gambling game, Yeung and Sanfey (2004) asked participants to choose between cards that were associated with monetary gains and losses of variable magnitude. They found that P300 was sensitive to reward magnitude but insensitive to reward valence.

With respect to the informative value of feedback, imaging studies have found that negative feedback containing different informative values activates different brain areas in rule learning. When negative feedback indicates that a rule is incorrect and participants need to shift the task set, the lateral prefrontal cortex, ACC, caudate nucleus, and parietal cortex are activated (Konishi et al., 1999; Monchi et al., 2001; Wilmsmeier et al., 2010). The dorsal lateral prefrontal cortex is more active following negative feedback that is informative for correct behavior in the next trial (Zanolie et al., 2008).

Previous ERP studies seldom directly investigated the informative value of feedback. First, in some tasks, such as the gambling task (Hajcak et al., 2006; Li et al., 2010) and guessing task (Baker and Holroyd, 2008; Lukie et al., 2014), the feedback could not be used to adjust behavior or improve learning. Second, in some tasks, although the feedback seemingly contained some useful informative value, the information cued by the feedback was ambiguous. For example, in the probability learning task (Cohen and Ranganath, 2007; Bellebaum and Daum, 2008; Chase et al., 2010), the informative value of feedback has been suggested to be related to P300. However, it should be noted that although participants could rely on the feedback information to keep or change their behavioral strategy, the informative value was not clear when the feedback was presented. That is, negative feedback that appeared once in a trial did not necessarily indicate that the rule had changed; the participants could be sure that the rule had been changed only when the negative feedback was displayed in more trials. Third, in the Wisconsin Card Sorting Test (WCST) and its modified version, the feedback contains both valence and informative value. Relevant studies revealed that negative feedback evoked a larger P300 than positive feedback (Furumoto, 1991; Watson et al., 2006). However, existing studies did not separate valence and informative value of feedback.

Only a handful of studies have attempted to address the neural mechanism underlying the informative value of feedback (Barceló, 1999, 2003; Kopp and Lange, 2013; Lange et al., 2015). In the variants of the WCST, Barceló (1999) distinguished the perseverative errors and efficient errors. A perseverative error was defined as a failure to change category in the second trial of a WCST series after having received negative feedback from the previous trial. In contrast, an efficient error was defined as a shift to the wrong category in the second WCST trial and always led to a correct sort in the third trial. These two types of errors are both negative feedbacks, but they differ in informative value. Specifically, a perseverative error implies that the previous sorting rule that has been demonstrated as invalid is still used, and participants should shift to the new rules; however, an efficient error implies that participants detected the change of sorting rule and did shift to a new rule that is not the correct one. Barceló (1999) found that the perseverative error induced a larger P300, and the efficient error induced a larger N1 in the parieto-occipital scalp and a larger P2 in the frontal region. In another study (Barceló, 2003) in which feedback only contained informative value, feedback that signaled the set-shifting induced the P300, which is sensitive to the number of rules held in memory. Both studies of Barceló (1999, 2003) suggested that P300 is sensitive to the informative value of negative feedback.

Similarly, Kopp and Lange (2013) demonstrated that participants could find the sorting rule in a task only when negative feedback was presented twice, and the second negative feedback induced a larger P300 than the first negative feedback. This study attempted to separate valence and informative value of feedback. However, the typical components associated with valence of feedback, such as N2 or FRN, were not observed possibly due to the added cues with different explicitness (e.g., “repeat,” “switch,” “shape,” “color,” or “number”). That is, when the cues were added in the rule shifting task, participants would use the cues to respond to stimuli and adjust behavior. Consequently, the role of feedback is weakened, reflecting the absence of FRN. In another study, Lange et al. (2015) compared informative feedback and redundant feedback. They found that the informative feedback elicited a larger P300 than the redundant feedback. However, the feedback (i.e., “switch” or “repeat” cues after each match) in this study had informative value but no valence.

In brief, only two research groups attempted to address the neural correlates of informative value of feedback, and both suggested that processing of informative value is related to P300. However, Barceló (1999, 2003) focused on the informative value of negative feedback but not positive feedback. Lange and colleagues used negative and positive feedback, but they either weakened the function of feedback in a task (Kopp and Lange, 2013) or emphasized the informative value of feedback by using cues without feedback valence (Lange et al., 2015). The problems of separating the informative value of feedback from feedback valence and examining corresponding neural processes in a task without using cues have not been solved yet.

**FIGURE 1:**
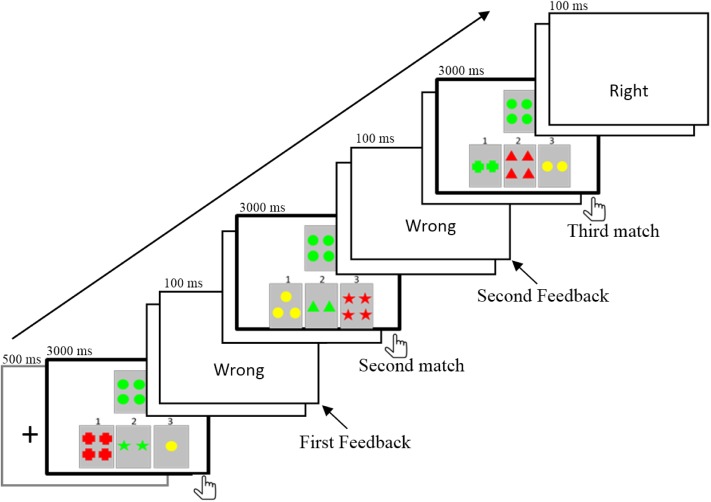
The procedure and the locations of feedback for each match.

The purpose of the present study was to elucidate the electrophysiological responses to the informative values of feedback in a rule acquisition task in which both the valence and the informative value of feedback were involved. We employed a segmented WCST (Wang et al., 2015). In the task, one target card and three reference cards were displayed on the screen (**Figure 1**). Participants were asked to match cards according to one rule (such as the same color), which could be acquired by trial and error in three consecutive trials. It was possible for participants to find the correct rule in their first attempt. Alternatively, some participants did not find the rule in the first try (received the negative feedback) but succeeded in the second or third try. That is, they might receive two feedbacks successively before finding the correct rule (Kopp and Lange, 2013). Therefore, feedback that participants received in separate attempts differed in informative value. The first negative feedback (1NF) informed the participants that the first formulated hypothesis or rule was incorrect. Participants then needed to consider the other two rules, and they were still not sure which one was the correct rule. In contrast, the second negative feedback (2NF) could rule out the second invalid rule and determine that the last rule was correct. The informative value of 2NF increased determinacy of the hypothesis, and the correct rule could be stored directly in working memory (Lange et al., 2015). Thus, compared with 1NF, 2NF was expected to induce a larger P300, which is often interpreted in terms of context-updating and updating working memory (Barceló and Knight, 2002; Kopp and Lange, 2013) or as processing relevant information about past events that could be used to modify future behavior (Müller et al., 2005).

In contrast to studies of Lange and his colleagues, the feedback in the present study had two components, valence and informative value. We hypothesized that the valence is the basis of informative value. That is, participants can learn the informative value of the feedback only when they know the correctness of their response. Therefore, we predicted that, in comparison with informative value, the effect of valence might occur during an earlier time window such as in FRN (Miltner et al., 1997; Gehring and Willoughby, 2002; Santesso et al., 2012).

Moreover, it has been found that in rule learning or a performance monitoring task, an enlarged frontal P2 was found after negative feedback as compared with positive feedback (Groen et al., 2007). The enhanced P2 has been explained as a reflection of motivational significance of negative feedback, and it is known to increase as task relevance of the stimuli increases (Potts, 2004; Gao et al., 2016). This suggests that participants might pay less attention to the stimuli that indicate less effort is needed in the current trial (Potts et al., 2006; San Martín et al., 2010; Pincham, 2014). We predicted that the first positive feedback (1PF) might evoke a decreased frontal P2 compared with 1NF, since 1PF indicates that the rule has been found so less effort is needed in the current trial. The frontal P2 evoked by 1NF might also be smaller than that of the second positive feedback (2PF), because when 2PF is displayed, participants had experienced a failure in matching. Therefore, they would carefully perform the second match and thus would be more likely to pay attention to the outcome of the second match.

## Materials and Methods

### Participants

Eighteen healthy right-handed volunteers (9 male, 9 female; age range: 18–25 years; mean age: 23 years) participated in this experiment. All participants reported normal or corrected-to-normal vision and no history of neurological or psychiatric impairments. All participants gave informed consent for the study before the experiment. Ethical approval for this study was obtained from the Jiangxi Normal University (Nanchang, China).

### Experimental Task

A segmented WCST (Grant and Berg, 1948; Milner, 1963; Wang et al., 2015) was used. One target card and three reference cards were displayed in the center of the screen (**Figure 1**). The reference cards were in the lower visual field and the target card was above the reference cards. The stimuli on each card consisted of three attributes including shape (cross, circle, triangle, and star), number (1, 2, 3, and 4), and color (red, green, yellow, and blue). Each reference card shared only one perceptual attribute with the target card. There were three matches in one trial. The target card was fixed, but the reference cards changed after each match. Participants had to match the target card to one of the three reference cards based on a hidden rule that linked with a shared perceptual dimension (e.g., shape, color, or number). Participants were explicitly instructed that there were three rules in the task. Each trial started with a fixed cross lasting for 500 ms. Then, a target card and three reference cards were displayed in the center of the screen. The participant was asked to choose one of three reference cards by pressing button 1, 2, or 3. The length of matching time was determined by each participant’s response time. They had to make a choice within 3000 ms; otherwise, “No response” was presented on the screen. After responding, there was a random interval of blank screen that lasted 800–1000 ms. The feedback was displayed for 100 ms, which was followed by a blank screen for 1000 ms. Then, the target and new reference cards appeared, and participants had to perform a second match. When the third match was finished, there was a 1000 ms blank screen. The rule was constant within each trial and varied randomly between trials. Before the experiment, the experimenter explained the task procedure to the participants in detail, and participants were required to perform a practice session until they clearly understood the task.

After each match, the feedback that participants received was based on their responses. For the data analysis, we only included the three types of trials in which participants correctly acquired the rule after three matches (see **Table 1**); the error trial (less than 4%) in which participants did not seriously perform the task were not included in the final analysis. The task was composed of 5 blocks, and each block contained 56 trials, which yielded a total of 280 trials. For all 18 participants, a total of 4737 trials eventually acquired the rule. Among the 4737 trials, participants received 1560 1PFs and 3177 1NFs after the first match. Among the 3177 1NF trials, participants received 1575 2PFs and 1602 2NFs after the second match. For each participant, the mean number of trials for 1PF, 1NF, 2PF, and 2NF were 87 (range, 75–93), 177 (158–191), 88 (80–97), and 89 (79–98), respectively. After artifacts were removed during ERP analysis, each participant retained approximately 50 valid trials per condition.

**Table 1:** Three types of trials in which participants acquired the rule successfully.

1st feedback	2nd feedback	3rd feedback	Trial type
NF	NF	PF	1
	PF	PF	2
PF	PF	PF	3


*Underlined red letters denote the four conditions analyzed in the present study*.

### Electrophysiological Recording and Analysis

The electrophysiological responses were recorded by active electrodes attached to an electrode cap (Brain Products GmbH, Munich, Germany) with a 64-channel EEG recording system. The online reference electrode was placed on FCz, and a ground electrode (AFz) was placed on the medial aspect of the frontal region. The vertical electrooculogram (VEOG) was recorded with electrodes placed above and below the right eye. Electrode impedances were kept below 10 kΩ. The EEG and EOG were amplified using a 0.01–100 Hz band-pass filter and continuously sampled at 500 Hz/channel. Trials with EOG artifacts (mean EOG voltage exceeding ± 80 μV) and those contaminated with artifacts due to amplifier clipping, bursts of electromyography (EMG) activity, or peak-to-peak deflection exceeding ± 80 μV were excluded.

Data were collected continuously and analyzed offline using Brain Vision Analyzer software (Brain Products GmbH, Munich, Germany). ERPs were re-referenced algebraically to the average of the left and right mastoids. Frequencies lower than 0.3 Hz (24 dB) or higher than 35 Hz (24 dB) were digitally filtered from the ERPs. The analysis epoch for the ERP was 1200 ms and time-locked to the feedback onset including a 200-ms pre-stimulus baseline. Amplitude was measured relative to feedback onset. The following 15 electrode sites were chosen to test the effects of condition, laterality and frontality (frontal: F3, Fz, F4; frontocentral: FC3, FCz, FC4; central: C3, Cz, C4; centroparietal: CP3, CPz, CP4; parietal: P3, Pz, P4).

Because there was no NF for the third match, we only analyzed the first and second feedback. P2 (150–200 ms) and FRN (250–350 ms) were elicited by all of the conditions, and the effects of conditions were mainly reflected in the frontal and central scalp. The mean amplitudes of these two components were analyzed using a 2 order (first, second) × 2 valence (positive, negative) × 3 laterality (left, middle, right) × 3 frontality (frontal, frontocentral, central) analysis of variance (ANOVA) with repeated measurements. P300 (350–550 ms) was elicited by all of the conditions, and the effects of conditions were reflected at all electrode sites. The mean amplitudes of this component were analyzed using a 2 order (first, second) × 2 valence (positive, negative) × 3 laterality (left, middle, right) × 5 frontality (frontal, frontocentral, central, centroparietal, parietal) ANOVA with repeated measurements. For all analyses, the *p*-values were corrected when necessary using the Greenhouse–Geisser method.

## Results

### Behavioral Results

**FIGURE 2:**
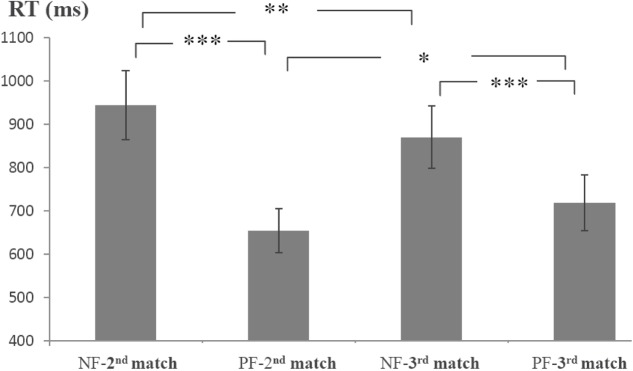
Average reaction times (RTs) for the 2nd and 3rd matches preceded by different feedback. NF-2nd match denotes the second match preceded by a negative feedback, and PF-2nd match denotes the second match preceded by a positive feedback. NF-3rd match denotes the third match preceded by a negative feedback, and PF-3rd match denotes the third match preceded by a positive feedback. Error bars refer to standard error of the mean. ^∗∗∗^*p* < 0.001; ^∗∗^*p* < 0.01; ^∗^*p* < 0.05.

Because the first and second feedback informatively affected the second and third matches, respectively, the average RTs of the second and third matches were analyzed (**Figure 2**). The RTs were submitted to a 2 order (first, second) × 2 valence (positive, negative) ANOVA with repeated measurements. There was a main effect of valence [*F*(1,17) = 66.79, *p* < 0.001, $\eta_{p}^{2}$ = 0.80]. The effect of order did not reach significance (*p* = 0.79). The interaction of order × valence was significant [*F*(1,17) = 11.44, *p* < 0.01, $\eta_{p}^{2}$ = 0.40]. Simple effect tests (Bonferroni corrected) indicated that the NF-2nd match (i.e., the second match preceded by a negative feedback) was slower than the PF-2nd match (i.e., the second match preceded by a positive feedback) [*F*(1,17) = 53.38, *p* < 0.001, $\eta_{p}^{2}$ = 0.76]; the RT of the NF-3rd match was slower than that of the PF-3rd match [*F*(1,17) = 31.78, *p* < 0.001, $\eta_{p}^{2}$ = 0.65]. The PF-3rd match was slower than that of the PF-2nd match [*F*(1,17) = 4.62, *p* < 0.05, $\eta_{p}^{2}$ = 0.21], and the NF-2nd match was slower than the NF-3rd match [*F*(1,17) = 9.23, *p* < 0.01, $\eta_{p}^{2}$ = 0.35].

### ERP Results

#### P2 Component

**FIGURE 3:**
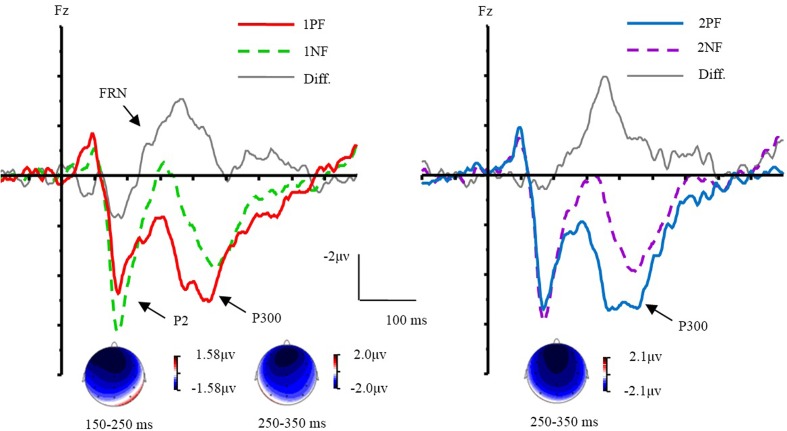
The effect of valence and topographical distribution of the difference at P2 and FRN. Left, ERPs evoked by the first feedback; Right, ERPs evoked by the second feedback.

**FIGURE 4:**
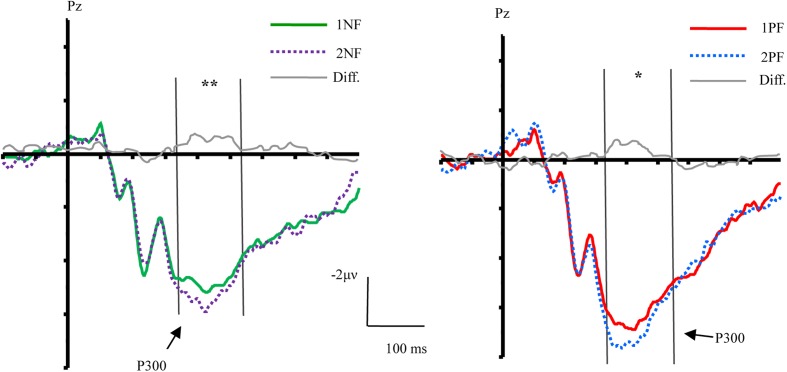
The effect of order and topographical distribution on the difference at the Pz electrode. Left, ERPs evoked by negative feedback; Right, ERPs evoked by positive feedback. ^∗∗^*p* < 0.01; ^∗^*p* < 0.05.

The ERPs evoked by different conditions were showed in **Figures 3**, **4**. Results of ANOVA revealed the main effect of valence [*F*(1,17) = 17.80, *p* < 0.01, $\eta_{p}^{2}$ = 0.51]. The factor of order did not yield a main effect (*p* = 0.42). The interaction of valence × laterality was significant [*F*(2,34) = 4.11, *p* < 0.05, $\eta_{p}^{2}$ = 0.20]. The simple effect analysis (Bonferroni corrected) indicated that negative feedback evoked larger P2 amplitudes than positive feedback at all electrode sites [*F*_left_ (1,17) = 18.16, *p* < 0.01, $\eta_{p}^{2}$ = 0.52; *F*_middle_ (1,17) = 18.21, *p* < 0.01, $\eta_{p}^{2}$ = 0.52; *F*_right_ (1,17) = 12.19, *p* < 0.01, $\eta_{p}^{2}$ = 0.42]. A three-way interaction of order × valence × frontality was significant [*F*(2,34) = 7.79, *p* < 0.01, $\eta_{p}^{2}$ = 0.31]. Further analyses were conducted by performing a 2 order (first, second) × 2 valence (positive, negative) ANOVA with repeated measurements at the frontal, frontocentral, and central sites, respectively. Over the frontal area, there was a main effect of valence [*F*(1,17) = 15.13, *p* < 0.01, $\eta_{p}^{2}$ = 0.47]. The factors of order (*p* = 0.40) did not yield significant effects. The interaction of order × valence was significant [*F*(1,17) = 16.29, *p* < 0.01, $\eta_{p}^{2}$ = 0.49]. The simple effect analysis revealed that the 1PF evoked a smaller P2 than the 2PF [*F*(1,17) = 6.16, *p* < 0.05, $\eta_{p}^{2}$ = 0.27]. The P2 evoked by 1PF was also smaller than that of 1NF [*F*(1,17) = 32.52, *p* < 0.001, $\eta_{p}^{2}$ = 0.66]. Over the frontocentral area, there was a main effect of valence [*F*(1,17) = 21.11, *p* < 0.001, $\eta_{p}^{2}$ = 0.55]. The factor of order did not yield a significant effect (*p* = 0.35). The interaction of order × valence was significant [*F*(1,17) = 8.99, *p* < 0.01, $\eta_{p}^{2}$ = 0.37]. The simple effect analysis revealed that the 1PF evoked a smaller P2 than the 2PF [*F*(1,17) = 5.51, *p* < 0.05, $\eta_{p}^{2}$ = 0.25]. The P2 evoked by 1PF was also smaller than that of 1NF [*F*(1,17) = 32.52, *p* < 0.001, $\eta_{p}^{2}$ = 0.66; *F*(1,17) = 29.53, *p* < 0.001, $\eta_{p}^{2}$ = 0.64]. For the central area, there were main effects of valence [*F*(1,17) = 14.84, *p* < 0.01, $\eta_{p}^{2}$ = 0.47]. The factor of order did not yield a significant effect (*p* = 0.56). The interaction of order × valence was significant [*F*(1,17) = 5.37, *p* < 0.05, $\eta_{p}^{2}$ = 0.24]. The simple effect analysis revealed that the 1PF evoked a smaller P2 as compared with 1NF [*F*(1,17) = 20.10, *p* < 0.001, $\eta_{p}^{2}$ = 0.54]. In brief, the valence effect was found readily, and the order effect was only found for the positive feedback during the P2 time window.

#### FRN Component

Results of ANOVA revealed the main effect of valence [*F*(1,17) = 32.74, *p* < 0.001, $\eta_{p}^{2}$ = 0.66]. The factor of order did not yield a main effect (*p* = 0.25). The interaction of valence × laterality was significant [*F*(2,34) = 6.60, *p* < 0.01, $\eta_{p}^{2}$ = 0.28]. The simple effect analysis indicated that negative feedback evoked more negative ERPs than PF from the left to right sites [*F*_left_(1,17) = 34.04, *p* < 0.001, $\eta_{p}^{2}$ = 0.67; *F*_middle_ (1, 17) = 30.32, *p* < 0.001, $\eta_{p}^{2}$ = 0.64; *F*_right_ (1,17) = 29.09, *p* < 0.001, $\eta_{p}^{2}$ = 0.63]. The interaction of valence × frontality was significant [*F*(2,34) = 4.32, *p* < 0.05]. The simple effect analysis indicated that negative feedback evoked more negative ERPs than positive feedback from the frontal to central sites [*F*_frontal_ (1,17) = 45.52, *p* < 0.001, $\eta_{p}^{2}$ = 0.73; *F*_frontocentral_ (1,17) = 27.58, *p* < 0.001, $\eta_{p}^{2}$ = 0.62; *F*_central_(1,17) = 23.99, *p* < 0.001, $\eta_{p}^{2}$ = 0.59]. We made an additional analysis to measure peak-to-peak of FRN, and the results of ANOVA revealed almost the same as the original results.

#### P300 Component

The main effect of valence was found [*F*(1,17) = 10.51, *p* < 0.01, $\eta_{p}^{2}$ = 0.38], and the main effect of order was significant [*F*(1,17) = 6.37, *p* < 0.05, $\eta_{p}^{2}$ = 0.27]. A 3-way interaction of order × valence × laterality was significant [*F*(2,34) = 5.41, *p* < 0.05, $\eta_{p}^{2}$ = 0.24]. Further analyses were conducted by performing a 2 order (first, second) × 2 valence (positive, negative) ANOVA with repeated measurements over the left, middle, and right areas, respectively. Over the left area, the main effect of valence [*F*(1,17) = 12.60, *p* < 0.01, $\eta_{p}^{2}$ = 0.43] and order [*F*(1,17) = 9.01, *p* < 0.01, $\eta_{p}^{2}$ = 0.35] were observed. There was no other interaction effect. Over the middle area, the main effect of valence [*F*(1,17) = 10.04, *p* < 0.01, $\eta_{p}^{2}$ = 0.37], and order [*F*(1,17) = 6.03, *p* < 0.05, $\eta_{p}^{2}$ = 0.26] were observed. There was no interaction effect. Over the right area, results of ANOVA revealed the main effect of valence [*F*(1,17) = 8.41, *p* < 0.05, $\eta_{p}^{2}$ = 0.33]. The factor of order did not yield a significant effect (*p* = 0.082). There was no interaction effect.

## Discussion

The purpose of the present study was to investigate the electrophysiological responses to informative values of feedback in a segmented WCST. The behavioral results revealed that the RTs for the NF-2nd match were longer than those for the PF-2nd match, reflecting the process of extracting and using the informative value from the negative feedback. The NF-2nd match was slower than the NF-3rd match, implying that less useful informative value was extracted from the 1NF match than from the 2NF match. Specifically, after rejection of the first rule, the informative value of 1NF did not tell the participants which of the remaining two rules was correct. Thus, they hesitated during the second match. In contrast, when two rules were rejected in succession, participants obtained useful informative value from the negative feedback. That is, they could be sure that the last rule was correct. Accordingly, the RT of the third match was shorter. RTs for the PF-3rd match were longer than those of the PF-2nd match. The longer RTs for the PF-3rd match may have been due to the additional process of inhibiting the rules rejected about 5 s ago.

Electrophysiological results revealed that the effect of feedback valence was reflected in wider epochs including P2, FRN, and P3, while the effect of order was reflected mainly in the P300 time window and in the P2 time window for positive feedback. These findings imply that there was a temporal dissociation between valence and informative value of feedback. The finding of a general FRN effect replicated the main finding of previous studies (Cohen and Ranganath, 2007; Eppinger et al., 2009; Bismark et al., 2013). The effect of order was not observed in the FRN time window, suggesting that the valence rather than the informative value of feedback was processed in this time window (Pfabigan et al., 2010; Borries et al., 2013; Peterburs et al., 2015).

The informative value of feedback, which was defined by the order effect, was reliably observed during the P300 time window. This result is in line with previous studies, which suggested that the P300 amplitude correlates with the amount of information that can be extracted from the feedback (Donchin and Coles, 1988; Johnson, 1988), and that the more information that can be extracted from the feedback, the more positive the P300 amplitude is (Barceló et al., 1997; Bellebaum and Daum, 2008; San Martín et al., 2010).

In a rule learning task, it is important to correctly process the negative feedback for behavior adjustment (Barceló, 1999; Kopp and Lange, 2013). Participants who failed in extracting the informative value of negative feedback would receive a lower score in the rule learning task (Du and Li, submitted). In the present study, compared with 1NF, the informative value of 2NF informed the participants that they were closer to the final answer. Specifically, the informative value of 1NF was to eliminate one rule. During the second match, participants needed to choose one of two remaining rules, but they were uncertain about which one was correct. In contrast, 2NF combined with 1NF was more useful in determining the right answer. When participants ruled out another rule after receiving 2NF, there was only one rule left. Participants could clearly know the correct answer. Therefore, the informative value of 2NF was greater than that of 1NF, resulting in a larger P300 than 1NF. This finding is consistent with Kopp and Lange (2013), who found that the second negative feedback induced a larger P300 than the first negative feedback. Moreover, the present study found that the informative value was mainly processed in the left sites, which is consistent with findings from previous imaging studies that revealed left laterality in updating of cognitive set (Rogers et al., 1998; Konishi et al., 2002; Stuss and Alexander, 2007; Ko et al., 2008). However, we should acknowledge that laterality in ERP studies is not the same as laterality in imaging studies due to the lower spatial resolution of ERP methods. Future imaging studies are needed to investigate laterality of processing of the informative value of feedback.

As mentioned above, previous studies did not investigate the neural correlates of informative value of positive feedback, possibly due to the difficulty in detecting the neural response to the unchanged cognitive set during the presentation of positive feedback. The core informative value of positive feedback is informing participants to maintain the selected rule in working memory (Monchi et al., 2001; Watson et al., 2006), but there might some difference in the processing of informative value of positive feedback at different stages of rule learning. Based on this presumption, we examined the order effect on positive feedback and clearly observed its modulation on the P300 component. Compared with 1PF, 2PF evoked a larger P300. Regardless of whether it was the first or second PF, the rule confirmed by PF was linked with one of three perceptual dimensions. Presumably, the information provided by 1PF should not differ from that of 2PF. The only difference is that none of the rules has been rejected before 1PF, whereas a rule has been rejected before 2PF. It is possible that the order effect for PF was caused by the process of inhibiting the old rule during the presentation of 2PF (De Bruijn et al., 2008; Smith et al., 2008). Hence, we inferred that although the main informative value of the two types of positive feedback is to keep the selected rule in working memory, when 2PF appears, participants should additionally inhibit the invalid rule that was selected during the first match. The additional inhibition for 2PF might be reflected by the increased P300 (Roberts et al., 1994; Xie et al., 2017).

It is necessary to note that the amplitude difference of P300 between negative and positive feedback might also be associated with processing of informative value of the two types of feedback. Regardless of the order of the feedback, positive feedback (1PF or 2PF) elicited a larger P300 than negative feedback (1NF or 2NF), possibly reflecting the difference in processing different informative values of different types of feedback. That is, the informative value of the positive feedback guides participants to maintain the confirmed rule in working memory, and the informative value of the negative feedback guides participants to inhibit the invalid rule and shift to the new rules. However, the present study found both a valence effect and an order effect during P300. The order effect in this time window exactly demonstrates that the informative value of feedback is processed in P300, but we cannot rule out the possibility that the valence of feedback is still processed in this time window (Hajcak et al., 2005; Yeung et al., 2005; Groen et al., 2007). Accordingly, the amplitude difference of P300 between negative and positive feedback was associated with both the valence and informative value difference between negative and positive feedback.

Finally, the present study found that the early attentional process is not the same for different kinds of informative value of feedback (Barceló, 1999), which was observed in the frontal P2 component. The P2 component has been interpreted as an index of selective attention or identification of perceptual representations (Potts et al., 2006; San Martín et al., 2010; Pincham, 2014) and was smaller for positive feedback as compared with negative feedback in the rule learning task (Groen et al., 2007). In the present study, the P2 evoked by 1PF was smaller than that of 1NF and 2PF in the frontal and frontocentral regions. This difference may have been due to paying less attention to 1PF than to 2PF. When 2PF was displayed, participants had experienced a failure in matching. Therefore, they carefully performed the second match and would have been more likely to pay more attention to the outcome of the second match. However, 1PF indicated that participants luckily found the rule and that less effort would be needed in the current trial (Potts, 2004; Gao et al., 2016). In addition, Barceló (1999) found that there were differences in the frontal P2 for the different kinds of negative feedback. However, in the present study, differences in the frontal P2 were found with the positive feedback but not the negative feedback. Future studies are needed to explore the different attentional processes of feedback that has the same valence but different informative values.

Despite these findings, there are several limitations to this study. The first limitation is that only 18 participants were examined in this study. The conclusion draw here might be not strong enough. Future study is needed to confirm the conclusion by using a larger number of participants. The second limitation is about the manipulative definition of informative value. We examined the different informative value of different feedbacks by comparing the order of the presentation of the feedback. The readers are suggested to cautiously understand the results by considering the order effect of feedback. Future research may address this issue by varying the amounts of informative value of feedback at the same order.

## Conclusion

We used a segmented WCST to study brain potential associated with the informative value of feedback by disassociating the feedback valence. The ERP results indicated that the valence was processed in a relatively wider epoch including P2, FRN, and P300. In contrast, the informative value of negative and positive feedback was primarily processed in the P300 time window. After experiencing an error, the informative value of the subsequent positive feedback was attended early in the frontal P2 time window.

## Author Contributions

Conceived and designed the experiments: FL and BC. Performed the experiments: JW. Analyzed the data: JW and BD. Wrote the paper: FL and JW.

## Conflict of Interest Statement

The authors declare that the research was conducted in the absence of any commercial or financial relationships that could be construed as a potential conflict of interest.

## Footnotes

1 Feedback valence occur in a long-time window including FRN and P300, particularly in the paradigm used here. In other paradigms (Oliveira et al., 2007; Ferdinand et al., 2012; Ferdinand and Opitz, 2014), the FRN is not correlated to feedback valence but sensitive to other cognitive components such as expectancy. In the present study, we presented subjects either positive feedbacks or negative feedbacks for their responses, and we did not manipulate the expectancy. So, it is inappropriate to use the label “expectancy.” Therefore, we used the label “valence”" to indicate whether the feedback is positive or negative in this communication.
